# The use of endoluminal techniques in the revision of primary bariatric surgery procedures: a systematic review

**DOI:** 10.1007/s00464-020-07468-w

**Published:** 2020-02-28

**Authors:** Yan Mei Goh, Nicole Ellen James, En Lin Goh, Achal Khanna

**Affiliations:** 1 Imperial College London, London, UK; 2 Department of General Surgery, Milton Keynes University Hospital, Milton Keynes, UK; 3 Oxford University Hospitals NHS Foundation Trust, Oxford, UK

**Keywords:** Endoluminal techniques, Revision surgery, Bariatric surgery

## Abstract

**Background:**

Weight regain following primary bariatric surgery is attributed to anatomical, behavioural and hormonal factors. Dilation of the gastrojejunal anastomosis is a possible cause of weight regain after roux-en-Y gastric bypass (RYGB). However, surgical revision has significant risks with limited benefits. Endoluminal procedures have been suggested to manage weight regain post-surgery. This systematic review aims to assess efficacy of endoluminal procedures.

**Methods:**

Studies where endoluminal procedures were performed following primary bariatric surgery were identified. Main outcome measures were mean weight loss pre- and post-procedure, excess weight loss, recurrence rates, success rates and post-procedure complications.

**Results:**

Twenty-six studies were included in this review. Procedures identified were (i) endoluminal plication devices (ii) other techniques e.g. sclerotherapy, mucosal ablation, and Argon Plasma Coagulation (APC) and (iii) combination therapy involving sclerotherapy/mucosal ablation/APC and endoscopic OverStitch device. Endoluminal plication devices show greatest initial weight loss within 12 months post-procedure, but not sustained at 18 months. Only one study utilising sclerotherapy showed greater sustained weight loss with peak EWL (19.9%) at 18 months follow-up. Combination therapy showed the greatest sustained EWL (36.4%) at 18 months. Endoluminal plication devices were more successfully performed in 91.8% of patients and had lower recurrence rates (5.02%) compared to sclerotherapy and APC, with 46.8% success and 21.5% recurrence rates. Both procedures demonstrate no major complications and low rates of moderate complications. Only mild complications were noted for combination therapy.

**Conclusions:**

The paucity of good quality data limits our ability to demonstrate and support the long-term efficacy of endoluminal techniques in the management of weight regain following primary bariatric surgery. Future work is necessary to not only clarify the role of endoluminal plication devices, but also combination therapy in the management of weight regain following primary bariatric surgery.

**Electronic supplementary material:**

The online version of this article (10.1007/s00464-020-07468-w) contains supplementary material, which is available to authorized users.

The role of bariatric surgery has grown significantly over the past decade, with an additional 10,000 procedures performed per year from 2011 to 2015, and an increase of 20,000 procedures from 2015 to 2016 in the USA [1]. In particular, gastric bypass, sleeve gastrectomy, adjustable gastric banding and biliopancreatic diversion with duodenal switch are frequently performed. These procedures are associated with significant long-term weight loss as well as alterations in gut hormone production and metabolism that suppress appetite and promote satiety. However, weight regain following primary bariatric surgery remains an ongoing problem. It is estimated that clinically significant weight regain occurs in up to one-third of patients [2–6] who have undergone a Roux-en-Y gastric bypass (RYGB) or vertical banded gastroplasty (VBG).

There are several factors that weight regain post-RYGB can be attributed to; notably a combination of lifestyle, mental health, hormonal/metabolic and surgical factors. Thus, the need to understand and address these issues with patients in the pre- and post-operative stage is crucial in preventing the reemergence of obesity related comorbidities and impaired quality of life [7, 8]. Non-surgical management of weight regain following bariatric surgery requires the input of the multidisciplinary team. Despite this, a proportion of patients still experience weight regain following bariatric surgery [9]. Dilatation of the gastrojejunal anastomosis or the gastric pouch is a well-recognised post-operative occurrence. On the basis that gastric pouch size, distension and transit time following RYGB is a surgical mechanism for early satiety and weight loss, this post-operative event may reduce the restrictive and malabsorptive effects of RYGB. Surgical revision of the gastrojejunal anastomosis is controversial as most patients are exposed to major post-operative complications, higher readmission rates and morbidity [9] but do not achieve significant weight reduction [10, 11].

Hence, endoluminal revision procedures have been developed to address this gap. These techniques come in various forms: endoluminal plication devices and other techniques like sclerotherapy, mucosal ablation and argon plasma coagulation. Endoluminal plication devices work by taking superficial or full-thickness bites of the intraluminal pouch mucosa or at the gastrojejunal anastomosis. Sutures or clips are then deployed via endoscope. Meanwhile, other techniques like sclerotherapy, mucosal ablation and APC induce scarring at the gastrojejunal anastomosis thus reducing its size. As the number of patients undergoing bariatric surgery continues to grow, the need to consider endoluminal revision procedures becomes increasingly important. Thus, this systematic review aims to assess efficacy of endoluminal techniques that attempt to revise primary bariatric procedures.

## Methods

### Criteria for considering studies for this review

All published studies that utilised endoluminal or endoscopic techniques following primary bariatric surgery were evaluated. Inclusion criteria are as follows: (a) studies investigating patients who had undergone endoscopic procedures following a primary bariatric surgery procedure (b) weight regain after surgery (c) presence or recurrence of comorbidities (d) post-operative complications (e) presence of anatomical cause for weight regain. Exclusion criteria are as follows: studies that did not include revision surgery, endoluminal procedures used in the management of complications following primary surgery, articles that assess primary bariatric surgery, non-endoluminal interventions, review articles, studies not written in the English language, animal studies, comment, opinions or letters, case reports and technical articles with no evidence of patient follow-up post-procedure, and conference abstracts.

### Literature search

The following databases were searched: (a) Medline (1946—present) via OvidSP, (b) MEDLINE Epub ahead of print, in process and other non-indexed citations (latest issue via Ovid SP, last search 19th July 2019); (c) Ovid Embase (1947—19th July 2019). Additionally, all references of included articles were manually reviewed to identify additional studies. Three strings were utilised; these terms were “bariatric surg*.mp. OR metabolic surg*.mp. OR weight loss surg*.mp.”, “revision*”.mp., “endoscopic procedure.mp. OR endosco*.mp.” and truncated search terms using wild card character and “related articles” function were used to broaden search. The references of included articles were also hand-searched to identify any additional studies.

### Data extraction and outcome measures

Two independent reviewers (YMG, NEJ) screened all titles and abstract manually for inclusion. A third reviewer (ELG) was consulted in the case of a disagreement. Relevant data were entered into Review manager 5.4 (Cochrane Collaboration, Oxford, United Kingdom). The following data items were extracted: year of publication, country of origin, study design, number of participants, type of primary procedure, type of endoluminal procedure performed, patient demographics, mean time since initial procedure, selection criteria in each study, mean pre-revision weight and BMI, mean weight loss post-procedure, complications post-procedure, average length of procedure, average stoma diameter at the end of the procedure, excess weight loss, length of follow-up and number of successful endotherapy.

### Quality assessment

Studies were appraised for rigorousness in methodology using the Newcastle–Ottawa Quality Assessment Scale [12] and risk of bias assessed using the National Institute of Health (NIH) Quality Assessment Tool for Case Series Studies [13].

## Results

**Fig. 1:**
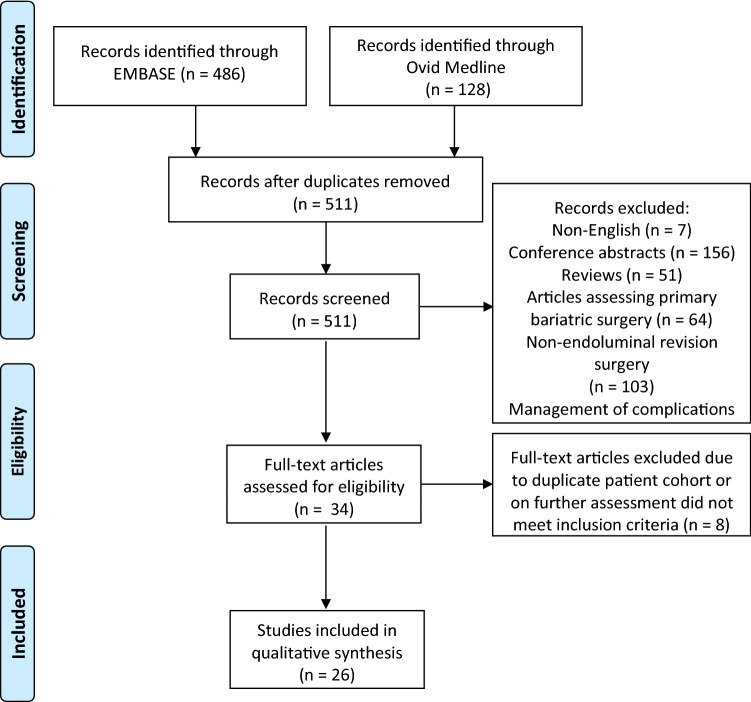
PRISMA chart of the study selection process

Twenty-six studies comprising a total of 1835 patients who had undergone endoluminal procedure following initial primary bariatric procedure were included in this study (Fig. 1). Endoluminal plication devices were used in 1087 patients, other techniques in 721 patients, and a combination of the two types of procedures in 27 patients. All studies were published over a period of twelve years from 2007 to 2019. There were eight prospective case series and one prospective multicentre randomised control trial. Of the 26 studies, 19 were performed in USA, one in Brazil, two in centres located in USA and Brazil, one in Belgium, one in France and one in Canada. Mean age of patients included in the review was 51.5 years old (range 22.0–71.4 years). The mean time since initial bariatric procedure was 86.7 months (range 12.0–222 months) (Table 1).

**Table 1:** Patient and study demographics

Study	Country	Study type	No	Primary Operation	Procedure	M:F	Mean age (years)	Mean time since initial procedure (months)	Selection criteria	Mean pre-revision weight (kg)	Mean pre-revision
											BMI (kg/m^2^)
Mikami et al. [14]	USA	Retro case series	39	RYGB	StomaphyX™	3:36	47.8 (29–64)		> 2 years post-op > 10% of nadir weight	108 (65.9–172.2)	39.8 (22.7–63.2)
Manouchehri et al. [26]	Canada	Pros case series	14	Vertical Banded Gastroplasty	StomaphyX™	1:13	47.3 ± 7.9	116.4 ± 73.2	Persistent WG	119.5 ± 25.9	43.4 ± 9.7
Ong'Uti et al. [15]	USA	Retro case series	27	RYGB	StomaphyX™	2:25	49 (44–54)	72 (60–96)	> 2 years post-op	103 (88.5–115)**	37 (32–40)
Goyal et al. (39)	USA	Retro case series	55	RYGB	StomaphyX™	1:53	49.6 (30–68)	68.4 (12–156)		96.6	36.1
Mullady et al. [29]	USA	Pros case series	20	RYGB	ROSE	1:19	48 (36–62)	63 (24–117)	WR/ no WL, satiety		36.7 (28,4–48.8)
Horgan et al. [18]	USA	Pros case series	116	RYGB	ROSE via Incisionless Operating Platform (IOP)	15:101	45.6 ± 8.7		> 2yrs post-op, > 50% EWL after RYGB	110.8 ± 20.5	39.9 ± 6.7
Ryou et al. [30]	USA	Pros case series	5	RYGB	ROSE	0:5	48 (41–55)	56 (24–76)	WR, satiety, dilated pouch/ GJA	100.4	36.3
Gallo et al. [17]	USA	Retro case series	27	RYGB	ROSE	2:25	49.2 ± 9.6 (26–68)	142.8 ± 51.6	> 50% EWL, sig WG 2 years post-op	106.2 ± 21.2**	40.6 (30–67)
Buttelmann et al. [31]	USA	Retro case series	8	RYGB	ROSE		48		Inadequate/ failure to lose weight		43.7
Thompson et al. [18]	USA	Retro case series	116	RYGB	IOP	15:101	46 ± 9		> 2yrs after RYGB	110.5 ± 20.5	39.9 ± 6.7
Heylen et al. [27]	Belgium	Pros case series	46	TVGB	OTSC-clip	19:75			> 10% WG 2yrs post-op, reappearing comorbidities, volume/ frequency of meals		32.8
Patel et al. [32]	USA	Retro case series	50	RYGB	EGJR	IST- 2:32	IST 48.6 ± 10.3	IST 115.2 ± 39.6	WR > 2yrs, stoma dilation > 15 mm	IST 114.5 ± 20.5	IST 41/7 ± 6/4
						PST- 2:14	PST 55.8 ± 10.8	PST 114 ± 42		PST 110.2 ± 22.6	PST 40.7 ± 8.7
Tsai et al. [22]	Switzerland	Retro case series	81	RYGB	EGJR (OverStitch device)	22:59	48.0 (26.8–71.4)	84 (12–222)	> 15 kg increase from nadir weight, 10 kg increase within 6–12 months post-op	127.1 (96–225)	44.7 (35.3–67)
Catalano et al. [33]	USA	Retro case series	28	RYGB	Sclerotherapy (sodium morrhuate)	10:18	41.1 (27–58)		Stoma size > 1.2 cm, WR after RYGB	112	
Loewen and Barba [34]	USA	Retro case series	71	RYGB	Sclerotherapy (sodium morrhuate)	4:67	45 (30–64)	34.8 (34.8–66	> 5% WG, inadequate WL < 50% EWL	98.1 ± 21.6**	35.5
Jirapinyo et al. [24]	USA	Pros case series	43	RYGB	Sclerotherapy (sodium morrhuate) (34)	3:31	47 ± 9	72 ± 60	> 1 yr post-op > 20% of nadir weight		
					TORe (9)	1:8	47 ± 13	84 ± 48			
Thompson et al. [21]	USA	Pros, multicentre RCT	50	RYGB	TORe	3:47	47.6 ± 9.46	58	BMI 30–60 at > 6 months post-op, Inadequate WL > 50% EWL/ WR > 5% EWL, GJA > 2 cm	101.5 ± 16.4	37.6 ± 4.9
de Moura et al. [39]	USA	Retro case report	1	RYGB	TORe	0:1	55	144		73.35**	27.9
Kumar and Thompson [37]	USA	Retro case series	59	RYGB	ST TORe	3:56	48.8 ± 1.1		Stoma diameter > 20 mm		40.4 ± 1.0
			59		FT TORe	15:44	49.9 ± 1.3				41.1 ± 1.3
Kumar and Thompson [36]	USA	Pros case series	150	RYGB	TORe (OverStitch device)	27:123	51.2 ± 0.8	103.2 ± 3.6	GJA > 15 mm	110.7 ± 2.2	40.1 ± 0.7
Jirapinyo et al. [25]	USA	Retro case series	25	RYGB	TORe (OverStitch device)	7:18	48 (34–69)	72 (24–120)	WR, GJA > 15 mm		43
Vargas et al. [23]	USA, Brazil	Retro case series	130	RYGB	TORe (OverStitch device)	16:114	47.12 ± 8.55	100.8 ± 57.4	WR		36.8 ± 6.84
Baretta et al. [20]	Brazil	Pros case series	30	RYGB	APC	4:26	42.83 (22–59)		> 18 months post-op, regain of > 10% of nadir weight, stoma diameter > 15 mm	121.77 ± 22.50	45.63 ± 7.63
Moon et al. [19]	USA, Brazil	Retro case series	558	RYGB	APC	103:455	40.9 ± 9.5	90 (60, 120)*	> 18 months post-op, regain of > 10% of nadir weight, satiety, size of GJ stoma > 15 mm	94.5 ± 18.6	34.0 ± 5.7
Riva et al. [35]	France	Retro case series	22	RYGB	Mucosal ablation + endoscopic suturing (OverStitch device) (11)	5:17	52.2 ± 11.7	106.8 ± 99.6	Sig WG > 50%	104.3 ± 27.4	42.4 ± 9.05
					Mucosal ablation + endoscopic suturing + sclerotherapy (OverStitch device) (11)					100.3 ± 27.0	42.4 ± 10.4
Eid [28]	USA	Retro case series	5	LSG	APC + endoscopic suturing (OverStitch device)	4:1	59.2 (48–63)	37.4 (32.2–48.2)	WR	110.25 (85.05–130.50)**	37

Study	Mean post-surgical weight (kg)					Mean post-revision BMI (kg/m2)					Complication post-procedure
	3 months	6 months	1 year	2 years	3 years	3 months	6 months	1 year	2 years	3 years	
Mikami et al. [14]	101.3	99.3	98								Minor: sore throat (87.1), epigastric pain (76.9)
Manouchehri et al. [26]	109.6 ± 24.4 (4 months)					39.8 ± 9.1 (4 months)					Minor headache, back pain
Ong'Uti et al. [15]	101.3	94.5	93.9 (81.6 ± 102)**			33 (29–36) (0 months)					
Goyal et al. (39)	92.9 (1 month)	92.8	94.9								Nil
Mullady et al. [29]											Minor: abdominal bloating, mild sore throats
Horgan et al. [18]		103									Mild: pharyhgitis 48 (41), Nausea/vomiting 14 (12), Abdo pain 13 (11) Moderate: superficial distal oesophagus tear 3(2.9)
Ryou et al. [30]	92.6					33.4					Nil
Gallo et al. [17]						39.2 ± 7		39.9 ± 10.1		37.7 ± 6.3	Nil
Buttelmann et al. [31]						40.6	40.7	39	38.9		Nil
Thompson et al. [18]			104.6								
Heylen et al. [27]						29.7		27.4			Mild: sore throat Moderate: 5(10.9) dysphagia (repeat OGD), 2 persistent dysphagia had endoscopic dilatation)
Patel et al. [32]											
Tsai et al. [22]	122.6	121.1	119.1								Nil
Catalano et al. [33]			92.1 (18 month)								Mild: 21 (75) post-injection pain. Moderate: 10 (35.7) shallow circumferential ulcers at stoma
Loewen and Barba [34]											
Jirapinyo et al. [24]											Pain 1, heartburn 1, hypertensive urgency 1, bleeding 1
											Pain 2
Thompson et al. [21]		95.1 ± 15.22									Mod: gastric mucosal tear, pulmonary oedema
de Moura et al. [39]		55.8**	49.5**				21.2**	18.8**			Nil
Kumar and Thompson [37]											Bleeding 1
											Bleeding 1
Kumar and Thompson [36]	101.1	100.1	100.2	90.7	91.5	36.6	36.3	36.3	36.8	36.7	Pain 6 (4.0), bleeding 5 (3.3), nausea 3 (2.0)
Jirapinyo et al. [25]											Hematemesis 1, delayed GI bleeding 1, nausea 4, severe emesis with torn stitches 2, stenosis of GJA 1
Vargas et al. [23]											Nausea 18 (14), Pain 23 (18), Oesophageal tear requiring endoscopic clipping 1 (< 1), balloon dilation of narrowed GJA after TORe 5 (4)
Baretta et al. [20]	83.29 (4 months)	78.87					31.14 ± 5.81				Severe stenosis (stoma diameter < 3 mm) 2, ulcers at stoma 10
Moon et al. [19]											Stenosis 9, GJ ulcer 3, vomiting 3, GJ leakage 2, melena 1
Riva et al. [35]							36	34			Minor: nausea and mild abdominal pain (44)
Eid [28]	100.08 (75.15–121.5)**	98.1 (72.9–119.25)**	99.09 (74.25–119.7)**					33.64 (26.7–44.9)			

Key: * median value, interquartile range, ** conversion from lb to kg (1 lb = 0.45)

*EWL* excess weight loss, *ROSE* restorative obesity surgery, endoluminal (ROSE) procedure, *EGJR* endoscopic gastrojejunal revision, *RYGB* Roux-en-Y gastric bypass, *LSG* laparoscopic sleeve gastrectomy, *OTSC-clip* over-the-scope clip, *IOP* incisionless operating platform, *TORe* sutured transoral outlet reduction, *ST* superficial-thickness, *FT* full-thickness, *APC* argon plasma coagulation, *Pros* prospective, retro: retrospective, *GJA* gastrojejunal anastomosis, *WR* weight regain, *WL* weight loss, *WG* weight gain, *EWL* excess weight loss

Twenty-five of the 26 included studies had clear selection criteria for all patients included in their study. These are as follows:

- Greater than 18 months following initial bariatric procedure [14–20]
- Weight regain or failure to lose sufficient weight [17, 19–35]
- Aged between 18 to 65 years old [16, 18, 32]
- Decreased satiety [19, 29, 30]
- Dilated gastrojejunal anastomosis and gastric pouch [19–21, 25, 30, 32, 33, 36, 37]
- Increased volume / frequency of meals [27]
- Reappearing comorbidities [27]
- BMI 30–60 kg/m^2^ greater than six months after RYGB [21]

One study [38] did not detail the inclusion nor exclusion criteria in patient selection.

### Endoluminal bariatric procedures

The endoluminal procedures identified were (i) endoluminal plication devices e.g. StomaphyX™, Restorative Obesity Surgery Endoluminal (ROSE) procedure, Incisionless Operating Platform (IOP), Over-The-Scope Clip (OTSC-Clip), e.g. sutured Transoral Outlet Reduction (TORe), Endoscopic Overstitch device and Endoscopic Gastrojejunal Revision (EJGR) and (ii) other techniques e.g. sclerotherapy, mucosal ablation, and Argon Plasma Coagulation (APC). Initial bariatric procedures performed were the roux-en-Y gastric bypass (RYGB), transected vertical gastric bypass (TVGB), vertical banded gastroplasty (VBG) and laparoscopic sleeve gastrectomy (LSG) (Tables 1,2).

**Table 2:** Roux-en-Y Gastric Bypass (RYGB)

Study	Procedure	Combination therapy (Y/N)	Av. procedure length (mins)	Av. stoma diameter at end of procedure (mm)	% Excess weight loss					
					1 week	1 month	2 months	3 months	6 months	12 months
Mikami [14]	StomaphyX™	N	35 (16–62)		(2 weeks) 7.4	10.6	13.1	13.1	17.0	19.5
Ong-Uti [15]	StomaphyX™	N		20 (20–30)	(2 weeks) 24			33	47	20
Goyal [38]	StomaphyX™	N	24.1 (10–55)	12.8	7.3 ± 7.1	11.6 ± 12.1			11.5 (17.9)	
Mullady et al. [29]	ROSE	N	103 (50–154)			5.8 kg*		8.8 kg**		
Horgan et al. [16]	IOP	N	87	11.5					21.5 ± 15.3	
Ryou et al. [30]	ROSE	N	80 (60–100)			4.2 kg**		7.8 kg**		
Gallo et al. [17]	ROSE	N	77 ± 30	8 ± 4				8.9	9.3	8
Buttelmann [31]	ROSE	N						3.9***	4.1***	5.4***
Thompson [18]	IOP	N	87	11.5						14.5 ± 3.1
Patel 2017 [32]	EGJR	N	IST 50.4 ± 25.3	IST 6.6 ± 2.2		6 weeks* 15 (9–22)		19 (9–27)*	13 (5–32)*	10 (− 3.2 to 23.1)
			PST 42.9 ± 18.1	PST 4.8 ± 1.8						
Tsai [22]	EGJR	N	17.2 (12–33)	6 (4–14)				4.1**	5.8**	8.0**
Catalano [33]	Sclerotherapy	N	10 (8–15)	10.4						
Loewen and Barba [34]	Sclerotherapy	N								
Jirapinyo [24]	Sclerotherapy (sodium morrhuate)	N		21 ± 6				2.7 ± 5.5****	6.1 ± 6.8 (9 months)****	
	TORe			23 ± 6				10.4 ± 2.2****	12.3 ± 12.6 (9 months)****	
Thompson [21]	TORe	N	107 ± 182.9						15.9	
de Moura [40]	TORe	N		12					20**	14**
Kumar and Thompson [37]	ST TORe	N		6.9 ± 0.2					8.1 ± 2.5	9.1 ± 2.3
	FT TORe			7.1 ± 0.3					20.4 ± 3.3	18.9 ± 5.4
Kumar and Thompson [36]	TORe (OverStitch)	N		9.0 ± 0.2				25.0 ± 1.9	28.8 ± 2.7	24.9 ± 2.6
Jirapinyo [25]	TORe (OverStitch)	N	27 (7–80)	6 (3–10)				11.5**	11.7**	10.8**
Vargas [23]	TORe (OverStitch)	N							9.31 ± 6.7	20.2 ± 10
Baretta [20]	APC	N		8.40 ± 1.85						
Moon [19]	APC	N	(5–10)	14.0 ± 6.3					6.5**	7.7**
Riva [35]	OverStitch + suturing	Y	91 ± 72.4	9.05						
	OverStitch + sclerotherapy									

Study	% Excess weight loss						Recurrence rates n (%)	Definition of successful endotherapy	Number of successful endotherapy n (%)
	18 months	24 months	36 months	48 months	60 months	72 months			
Mikami [14]									
Ong-Uti [15]							3 (4.7%)		
Goyal [38]		4.3 ± 29.8					2 (3.6)–progressed to further procedure	1. Ability to reduce pouch and stoma size 2. Weight loss	35 (63.6)
Mullady et al. [29]								1. Ability to reduce stoma diameter and pouch length 2. Weight loss	17 (85)
Horgan et al. [16]								1. Ability to reduce stoma diameter and pouch length 2. Weight loss	112 (97)
Ryou et al. [30]								1. Ability to reduce stoma diameter and pouch length 2. Weight loss	5 (100)
Gallo et al. [17]		6.7	− 10.7	− 13.5	− 5.8	− 4.5			
Buttelmann [31]		5.5***							
Thompson [18]								1. Ability to reduce stoma diameter and pouch length 2. Weight loss	112 (97)
Patel 2017 [32]							IST 3 (8.8)		
							PST 0		
Tsai [22]									
Catalano [33]	19.9							1. Stoma size < 12 mm 2. Loss of > 75% of weight regained after initial weight loss	18 (64)
Loewen and Barba [34]							2nd session 35 (49), 3rd session 10(14), 4th session 1(1.4)		21 (29.6)
Jirapinyo [24]								Ability to reduce the GJ to < 12 mm	
Thompson [21]								Ability to reduce the GJ to < 10 mm	89.6%
de Moura [40]								Weight maintenance/ weight loss	24 (29.6)
Kumar and Thompson [37]									
Kumar and Thompson [36]		20.0 ± 6.4	19.2 ± 4.6						
Jirapinyo [25]								Ability to reduce the GJ to < 12 mm	25 (100)
Vargas [23]	8 ± 8.8 (18–24mths)						11 (8)—repeat EGD performed	Ability to reduce the GJ to < 10 mm	
Baretta [20]									
Moon [19]		8.3**							
Riva [35]	36.4								

Key: * median value, interquartile range, ** mean weight loss, *** mean BMI loss, **** mean %TBWL

*TBWL* total body weight loss, *EWL* excess weight loss, *ROSE* restorative obesity surgery, endoluminal (ROSE) procedure, *EGJR* endoscopic gastrojejunal revision, *RYGB* Roux-en-Y gastric bypass, *OTSC-clip* over-the-scope clip, *IOP* incisionless operating platform, *TORe* sutured transoral outlet reduction, *GJA* gastrojejunal anastomosis, *APC* Argon plasma coagulation

### Weight loss

Excess weight is defined as the difference between the patient’s actual weight and ideal weight. The percentage excess weight loss (EWL) is defined as the proportion of weight loss after endoluminal procedures divided by the difference of regained weight from nadir weight.

Revision surgery using endoluminal plication devices were performed in 18 studies post-RYGB [14–18, 21–25, 29–32, 36–39]. Results of these studies were analysed together. Of these, Stomaphyx™ was performed in three studies [14, 15, 38], ROSE in five studies [16, 17, 29–31], IOP in one study [18], TORe in seven studies [21, 23–25, 36, 39] and EJGR in two studies [22, 32]. These procedures were performed a mean of 91.2 months (12.0–222 months) after RYGB. Mean pre-revision weight was 105.6 kg (65.9–225 kg). Mean weight loss (6.27 kg) was greatest within the first 3 months post-procedure. This weight loss was sustained for up to two years after the revision endoluminal procedure. Post-procedure BMI within the first three months after the revision procedure had decreased by a mean of 7.61%, but there are insufficient data to comment on mean post-procedure BMI after two years. Mean EWL was sustained at 19.3% six months following the initial procedure. However, this was not maintained in patients two years post-procedure (EWL 10.3%).

There were two studies in which endoluminal procedures were performed in patients following VBG and TVGB, respectively [26, 27]. One study utilised the StomaphyX™ for revision of VBG [26]. The authors demonstrated a weight reduction of 9.9 kg at four months post-revisional procedure, with a decrease in BMI of 3.6 kg/m^2^ (8.28% weight loss) over the same time period [26]. The other study reported a mean decrease in BMI of 3.1 kg/m^2^ (9.45% weight loss) following the use of the OTSC-clip at 3 months post-revisional procedure in a group of TVGB patients [27]. This was sustained at 7.01% at 12 months post-revisional procedure. On review of both papers, neither study had reported the EWL following endoluminal revision surgery.

**Fig. 2:**
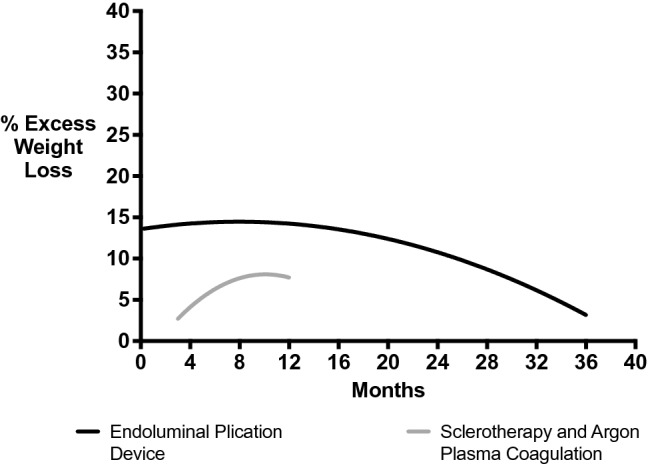
Graph demonstrating percentage EWL over time in endoluminal plication devices and others (sclerotherapy and APC)

All endoluminal plication devices post-RYGB showed a mean overall decrease in EWL over the first three months of 13.9% [14–18, 21–25, 29–32, 36–39]. This EWL was sustained at 13.7% at the 12-month follow-up (Fig. 2). Following this, the percentage EWL after 12 months post-procedure is demonstrated to show a steady decline to 8.5% 36 months post-procedure. Endoluminal plication devices were shown to be successful in 91.8% of patients in studies which provided data. Definitions of success in the various procedures are outlined where data are available (Table 2). These include the ability to reduce the diameter of the gastrojejunal stoma and pouch length [16, 18, 21, 23, 25, 29, 30, 38], as well as weight loss post-procedure [16, 18, 29, 30, 38, 39]. Recurrence rates and need for further procedure following endoluminal plication devices were 5.02%.

Meanwhile, the other techniques used post-RYGB i.e. sclerotherapy and APC showed a much lower weight loss compared to endoscopic plication devices with a 3.87% EWL three months post-procedure [19, 20, 24, 33, 34]. A 19.9% EWL at 18 months post-procedure is reported in Catalano et al.’s study utilising sclerotherapy [33], which is greater than the EWL (13.0%) in endoluminal plication devices. Sclerotherapy and APC were shown to be less successful in 46.8% of patients when compared to utilisation of endoluminal plication devices (91.8%), and had higher recurrence rates (21.5%).

### Complications

Six studies reported no complications following procedures involving endoluminal plication devices [17, 22, 30, 31, 38, 39]. Minor complications reported were abdominal pain (22.5%), sore throat (49.4%), device failure (3.1%), nausea and vomiting (11.0%). A greater range of moderate complications was reported. Specifically, 9.02% of patients reported moderate complications of mucosal tear or damage, 4% reported haematemesis, 2.7% reported bleeding and 10.9% had dysphagia following endoluminal plication. No major complications were reported by any study utilising endoluminal plication devices.

Studies utilising other techniques of sclerotherapy and APC reported minor complication of post-injection pain, abdominal pain and nausea (60%) and moderate complication of mucosal ulceration (35.7%). No major complications were reported.

### Outcomes of combination therapy

Only two studies assessed the use of combination therapy [28, 35]. Riva et al.’s study [35] involved a combination of mucosal ablation and endoscopic suturing using the OverStitch device or mucosal ablation, endoscopic suturing and sclerotherapy, performed following RYGB in 22 patients. Mean pre-revision weight and BMI were 102.3 kg and 42.4 kg/m^2^, respectively. Mean post-revision BMI was reduced by 15.1% at 6 months, and this was increased to 19.8% at 1 year. However, no mean weight or excess weight loss following the revision procedure was reported. In terms of complications, only abdominal pain and nausea were reported (44%).

The other study by Eid [28] consisted of five patients, in whom a combination of APC and endoscopic suturing (OverStitch device) was performed. Mean pre-revision weight was 110.3 kg and mean pre-revision BMI 37 kg/m^2^. In this group of patients, greatest weight loss was noted 6 months post-procedure (11.0%) and this reduced slightly to 10.1% at 1 year. This is consistent with a fall in 9.1% of mean post-revision BMI measured at 1 year. No complications were reported.

### Quality of included studies

All studies were evaluated for risk of bias using the Newcastle–Ottawa Scale (NOS), which allows for a maximum of eight points, and NIH Quality Assessment Tool (Table 3). A score of 6 or more on the NOS is rated “good”, while the NIH tool judges each study to be of “good”, “fair” or “poor” quality. Both the NOS and NIH tool assess risk of bias in the selection of cases, outcome assessment and duration of follow-up. Additionally, the NOS assesses the representativeness of the exposed cohort and adequacy of follow-up. Of the 25 observational studies, two studies achieved a score of 7, seven were scored 6, nine rated 5, five scored 4 and two scored 3 on the NOS (Table 3, Supplementary Table 1).

**Table 3:** Summary of quality and risk of bias assessment using the Newcastle–Ottawa scale and National Institute of Health quality assessment tool for case series studies

Study	Newcastle–Ottawa scale	National Institute of Health quality assessment tool	Overall
Mikami et al. [14]	4	Fair	Fair
Manouchehri et al. [26]	6	Good	Good
Ong’Uti et al. [15]	7	Good	Good
Goyal et al. (39)	7	Good	Good
Mullady et al. [29]	5	Fair	Fair
Horgan et al. [16]	6	Good	Good
Ryou et al. [30]	4	Poor	Poor
Gallo et al. [17]	4	Fair	Fair
Buttelmann et al. [31]	6	Good	Good
Thompson et al. [18]	5	Fair	Fair
Heylen et al. [27]	5	Fair	Fair
Patel et al. [32]	5	Fair	Fair
Tsai et al. [22]	6	Good	Good
Catalano et al. [33]	5	Good	Fair
Loewen and Barba [34]	4	Fair	Fair
Jirapinyo et al. [24]	6	Good	Good
de Moura et al. [39]	3	Poor	Poor
Kumar and Thompson [37]	5	Fair	Fair
Kumar and Thompson [36]	6	Good	Good
Jirapinyo et al. [25]	5	Fair	Fair
Vargas et al. [23]	5	Good	Fair
Baretta et al. [20]	4	Fair	Fair
Moon et al. [19]	5	Fair	Fair
Riva et al. [35]	6	Good	Good
Eid [28]	3	Poor	Poor

Twenty-three studies (92%) lacked a comparator group. In all studies, a satisfactory length of follow-up is defined as 12 months or longer—this criteria was met by 20 (80%) studies. Four (15.4%) studies were not awarded an “outcome” score on the Newcastle–Ottawa Scale due to high attrition rates. Eleven (42.3%) studies failed to report the rate of patient follow-up. Two studies in particular [14, 16] had very low follow-up rate (< 20%) at 12 months. The authors defined 12 months as the ideal duration of follow-up for post-procedural assessment of weight loss and complications following StomaphyX [14] and ROSE [16], respectively, in their selected patients, all of whom were at least 2 years post-RYGB. However, only 15.4% [14] and 11.2% [16] were successfully followed up for post-procedural evaluations at 12 months, and the authors did not elaborate the reason(s) behind loss to follow-up.

With regards to the NIH Quality Assessment Tool (Table 3, Supplementary Table 2), 12 (46.2%) studies were subjectively considered to be of “good” quality, while 11 (42.3%) were considered “fair” and three (11.5%) were rated “poor” in the risk of bias assessment. The studies rated “poor” lacked a clear definition for cohort selection and outcome measures, and either failed to describe the results or statistical methods used. Given the high heterogeneity of the studies included in this review, the results and conclusions should be interpreted with caution.

## Discussion

Weight regain is estimated to range between 5–7% [2, 4, 5] with higher failure rates (20–35%) in the superobese patients (BMI > 50) [6, 40, 41]. This systematic review addresses less invasive techniques for treatment of post-operative weight gain and associated short-term outcomes. The use of endoluminal plication devices in revisional surgery is associated with greater initial EWL and fewer complications compared to other techniques (i.e. sclerotherapy, APC) post-RYGB. More specifically, analysis of the included studies has shown successful EWL following the use of endoluminal plication devices in the first 12 months after revisional procedure. This EWL was, however, not well-sustained past 12 months. Greater procedural success and lower recurrence rates are seen in endoluminal plication devices compared to sclerotherapy and APC. Additionally, this review suggests that endoluminal plication devices are associated with lower rates of mild and moderate complications post-procedure compared to sclerotherapy and APC.

Due to the complex nature of weight regain, which involves an interplay between genetic, anatomical, physiological and behavioural factors [42, 43], there are different theories as to which factor is most predictive of treatment response. Excess weight loss (EWL) following revision surgery using endoluminal plication devices is likely to be due to anatomical reasons. Horgan et al. describes failure of maintenance of EWL to be due to loss of restriction attributed to the enlargement of the gastric pouch, dilatation of gastrojejunostomy and fistula development between gastric pouch and remnant of the stomach [16]. In this review, Vargas et al.’s study was focused on stoma size reduction, where the revision procedure (TORe) was considered successful if the stoma diameter was reduced to < 10 mm [23]. The authors achieved a mean of 70.4% reduction in stoma diameter post-procedure and a resultant mean EWL of 20.2% at 12 months, and 8.0% at 18 months [23]. Similarly, Jirapinyo et al. showed a mean reduction of 77.3% in stoma diameter post-procedure in their cohort of 25 patients post-procedure (TORe), with a peak mean weight loss of 11.7 kg at 6 months, which later decreased to 10.8 kg at 12 months [25]. The greater mean EWL in the initial 12 months following the use of endoluminal plication devices in patients post-RYGB which decreased in the ensuing months may be attributed to the lack of durability of endoscopic sutures in the long term [44]. Follow-up endoscopy after ROSE procedures showed that superior weight loss is associated with reduction in stoma size, with good durability of anchors and tissue fold for up to 12 months post-revision [16, 18]. These studies provide evidence that stoma size does influence weight loss post-revision surgery, where EWL is greatest in the first 12 months. However, long-term data past 18 months are not recorded for most studies utilising endoluminal plication devices post-RYGB in this review. Future studies, which include follow-up evaluations with endoscopy to verify the link between maintenance of stoma reduction and EWL, are warranted.

Another possible predictor of EWL following revision surgery is ghrelin levels post-procedure. The role of ghrelin in obesity in previous work appears to be significant, however, its exact mechanism requires further investigation [45, 46]. However, Dayyeh et al. demonstrated a decrease in ghrelin levels in a group of 33 RYGB patients post-sclerotherapy, contrary to what was observed following mechanical endoscopic suturing with endoluminal plication devices [42]. The authors postulated that ghrelin-producing cells were destructed as a result of sclerosis, hence modulating the neurohormonal signalling to the brain and other organs, altering satiety, food intake behaviours insulin secretion and energy expenditure [42, 47]. This alteration in neurophysiology may account for the greater sustained EWL over a longer period of time in sclerotherapy compared to endoluminal plication devices. This is because the latter predominantly depends on the reduction in GJ stoma diameter slowing down the activation of gastric wall mechanoreceptors, inhibiting the release of orexigenic gastric peptides such as ghrelin [42, 46–48], which may be less effective compared to a direct destruction of ghrelin-producing cells in sclerotherapy in inducing neurophysiological changes contributing to sustained weight loss.

Theoretically, the modulation in neurohormonal signalling may presumably be applied to APC, but the APC procedures that were utilised in two studies included in this review were aimed at reducing the diameter of the GJ stoma and, therefore, reinitiate weight loss in RYGB patients [19, 20]. Hence, these studies focused on the anatomical aspect of causes in weight regain, similar to that in endoluminal plication devices, although Moon et al. did demonstrate a sustained mean weight loss up to 24 months, longer than those noted in revisional procedures utilising endoluminal plication devices [19].

Furthermore, Manouchehri et al. has shown that endoluminal plication devices in revision surgery, specifically the StomaphyX™, can effectively contribute to weight loss in patients following VBG, with only minor complications experienced by patients [26], although sustained weight loss is not demonstrated due to limited duration of follow-up (3 months). However, the role of endoluminal plication devices is more skewed towards that in RYGB patients because VBG has largely been supplanted by RYGB as a primary bariatric surgery technique in recent years. Nevertheless, outcomes following endoluminal revisional techniques in VBG may still be of interest in a small cohort of patients experiencing weight regain requiring revisional surgery [49–51].

A previous meta-analysis by Vargas et al. has demonstrated the safe and efficacious use of TORe (OverStitch device) in revision surgery performed in RYGB patients [23]. The present review builds on this finding, and summarises qualitatively the evidence supporting greater long-term post-procedure weight loss when endoscopic suturing with OverStitch device is combined with sclerotherapy or APC, as shown by Riva et al. [35] and Eid [28]. Riva et al.’s study was aimed at investigating a possible additive effect of combined sclerotherapy and endoscopic suturing, where the induced fibrosis could enhance the durability of sutures [35]. Compared with sclerotherapy/APC (EWL 19.9%) or endoluminal plication device (EWL 12.9%) alone, combination therapy is shown to induce the greatest mean EWL of 36.4% at 18 months in a small study of five patients [28]. Although combination therapy appears to have some benefit in one study, this has not translated to a larger study of 22 patients.

This, compounded by the lack of clear description on patient selection and specification of outcomes, undermines the internal validity of the conclusions. This finding may suggest the potential of combination therapy in managing weight regain following primary bariatric surgery, however, there is currently insufficient evidence to support its superiority over endoluminal plication devices, and vice versa.

## Study limitations

The included studies exhibit some limitations, which must be considered when interpreting the findings of this analysis. Firstly, there is significant variation in primary bariatric procedure, endoluminal revision techniques, methodology of reporting, follow-up times, outcomes and complications. There are limited data on endoscopic revision procedures following sleeve gastrectomy. Given the rapid increase in use of sleeve gastrectomy in recent years, future research on the generalisability and applicability of endoscopic revision surgery in patients with sleeve gastrectomy will be necessary to overcome the inherent limitations of the currently available evidence. Moreover, the heterogeneity of the studies, especially with regards to the selection criteria of patients for revisional surgery, limits the statistical analysis of demographic and procedural variables that appeared to be predictive of maximal weight loss benefit.

Most series have small number of patients and some follow-up data were not available which imposes limits on our ability to make a meaningful conclusion. These high attrition rates could be attributed to a poor understanding of patients’ expressed needs, which is central to the development and delivery of effective longer term follow-up care following revision surgery. Studies have shown that patients who did not attend regular follow-up commonly described unmet perceived expectations as well as fear of disappointing the healthcare professional if they were unable to meet nutritional or physical activity targets set [52].

Additionally, these studies also lacked control of confounding factors including patients’ nutritional status, maintenance of diet and exercise, as well as important comorbid conditions such as type 2 diabetes mellitus. Future work may wish to explore the impact of additional routine follow-up addressing these behavioural issues and dietary and lifestyle modifications on maintenance of weight loss. Additionally, all of the studies were conducted in developed countries. These skewed study populations are unlikely to represent faithfully the true populations in less developed countries, thus the generalisability of these findings to the wider population in other parts of the world should be treated with caution. With the majority of studies being retrospective in design and the paucity of studies assessing long-term EWL of greater than 12 months following endoluminal procedures, the question whether endoluminal techniques can sustain long-term EWL still remains. Cohort studies or randomised controlled trials should be performed to not only clarify the role of endoluminal plication devices, but also combination therapy in the management of weight regain following primary bariatric surgery.

## Conclusion

Our study demonstrates the need for detailed discussion and tailoring of techniques and resources to the individual patient. Endoluminal techniques at present affords the patient an opportunity to alter their lifestyle and delay surgical revision or conversion to distal RYGB or biliopancreatic/duodenal switch procedures. However, the paucity of good quality data limits our ability to demonstrate and support the long-term efficacy of endoluminal techniques in the management of weight regain following primary bariatric surgery. However, we suggest that these techniques have an intermediate role in management of weight regain following bariatric surgery, delaying surgical revision or conversion to distal RYGB or biliopancreatic/duodenal switch procedures. Future work is necessary to substantiate the long-term role of endoluminal bariatric procedures in the management of this group of patients.

## Electronic supplementary material

Below is the link to the electronic supplementary material.

Supplementary file1 (DOCX 40 kb)

Supplementary file2 (DOCX 42 kb)
