# Supplementary material for: The use of endoluminal techniques in the revision of primary bariatric surgery procedures: a systematic review
Source: Surg Endosc. 2020 Feb 28;34(6):2410–28. doi: 10.1007/s00464-020-07468-w (PMC7214483; doi:10.1007/s00464-020-07468-w)
Supplement: Supplementary file 1 — Supplementary file1 (DOCX 40 kb) [file 464_2020_7468_MOESM1_ESM.docx]

Supplementary Table 1: Newcastle-Ottawa Scale for included studies

| **Study** | **Selection** | | | | **Comparability** | **Outcome** | | | **Score** |
| --- | --- | --- | --- | --- | --- | --- | --- | --- | --- |
|  | **Representativeness of exposed cohort** | **Selection of non-exposed cohort** | **Ascertainment of exposure** | **Outcome of interest not present at start** | **Comparable based on design or analysis** | **Assessment of outcome** | **Length of follow-up (>12 months)** | **Adequacy of follow-up (>70%)** |  |
| Mikami (2010) (14) | Truly representative | No description | Surgical record | Yes | No | No description | 12 months | 15.4% | **4** |
| Manouchehri (2011) (26) | Somewhat representative | Same | Surgical record | Yes | No | Record linkage | 12 months | No description | **6** |
| Ong’Uti (2013) (15) | Truly representative | Same | Surgical record | Yes | No | Record linkage | 12 months | 88.9% | **7** |
| Goyal (2013) (39) | Truly representative | Same | Surgical record | Yes | No | Record linkage | 18 months | 89.8% | **7** |
| Mullady (2009) (29) | Truly representative | Same | Surgical record | Yes | No | Record linkage | 3 months | No description | **5** |
| Horgan (2010) (16) | Truly representative | Same | Surgical record | Yes | No | Record linkage | 12 months | 11.2% | **6** |
| Ryou (2009) (30) | No description | No description | Surgical record | Yes | No | Record linkage | 3 months | Complete | **4** |
| Gallo (2016) (17) | Truly representative | No description | No description | Yes | No | Record linkage | 12 months | 40% | **4** |
| Buttelmann (2015) (31) | Truly representative | Same | No description | Yes | Yes | Record linkage | 12 months | No description | **6** |
| Thompson (2012) (36) | Truly representative | No description | Surgical record | Yes | No | Record linkage | 12 months | 65.2% | **5** |
| Heylen (2011) (27) | Truly representative | No description | Surgical record | Yes | No | Record linkage | 12 months | No description | **5** |
| Patel (2017) (32) | Truly representative | No description | Surgical record | Yes | No | No description | 12 months | 73.2% | **5** |
| Tsai (2019) (22) | Somewhat representative | Same | Surgical record | Yes | No | Record linkage | 12 months | No description | **6** |
| Catalano (2007) (33) | Truly representative | No description | Surgical record | Yes | No | Record linkage | 18 months | No description | **5** |
| Loewen (2008) (34) | Truly representative | No description | Surgical record | Yes | No | No description | 12 months | No description | **4** |
| Jirapinyo (2016) (24) | Truly representative | No description | Surgical record | Yes | No | Record linkage | 9 months | 70.0% | **6** |
| de Moura (2019) (40) | No description | No description | No description | Yes | No | No description | 6 months | Complete | **3** |
| Kumar and Thompson (2014) (38) | Somewhat representative | Same | Surgical record | Yes | No | No description | 12 months | No description | **5** |
| Kumar and Thompson (2016) (37) | Truly representative | No description | Surgical record | Yes | Yes | No description | 36 months | 92.7% | **6** |
| Jirapinyo (2013) (25) | Truly representative | No description | Surgical record | Yes | No | No description | 12 months | 78.3% | **5** |
| Vargas (2018) (23) | Truly representative | No description | Surgical record | Yes | No | Record linkage | 18 months | No description | **5** |
| Baretta (2015) (20) | Truly representative | No description | Surgical record | Yes | No | No description | 18 months | No description | **4** |
| Moon (2018) (19) | Truly representative | No description | Surgical record | Yes | No | Record linkage | 36 months | No description | **5** |
| Riva (2017) (35) | Somewhat representative | No description | Surgical record | Yes | No | Record linkage | 22 months | Complete | **6** |
| Eid (2017) (28) | No description | No description | No description | Yes | No | No description | 12 months | Complete | **3** |
