# Supplementary material for: The use of endoluminal techniques in the revision of primary bariatric surgery procedures: a systematic review
Source: Surg Endosc. 2020 Feb 28;34(6):2410–28. doi: 10.1007/s00464-020-07468-w (PMC7214483; doi:10.1007/s00464-020-07468-w)
Supplement: Supplementary file 2 — Supplementary file2 (DOCX 42 kb) [file 464_2020_7468_MOESM2_ESM.docx]

Supplementary Table 2: Risk of bias assessment using the National Institute of Health Quality Assessment Tool for case series studies

| **Study** | **1. Was the study question or objective clearly stated?** | **2. Was the study population clearly and fully described, including a case definition?** | **3. Were the cases consecutive?** | **4. Were the subjects comparable?** | **5. Was the intervention clearly described?** | **6. Were the outcome measures clearly defined, valid, reliable, and implemented consistently across all study participants?** | **7. Was the length of follow-up adequate?** | **8. Were the statistical methods well-described?** | **9. Were the results well-described?** | **Overall** |
| --- | --- | --- | --- | --- | --- | --- | --- | --- | --- | --- |
| Mikami (2010) (14) | No | Yes | Yes | NA | Yes | No | Yes | No | Yes | Fair |
| Manouchehri (2011) (26) | Yes | Yes | Yes | NA | Yes | Yes | Yes | Yes | Yes | Good |
| Ong’Uti (2013) (15) | Yes | Yes | Yes | NA | Yes | Yes | Yes | Yes | Yes | Good |
| Goyal (2013) (39) | Yes | Yes | Yes | NA | Yes | Yes | Yes | Yes | Yes | Good |
| Mullady (2009) (29) | Yes | Yes | Yes | NA | Yes | No | No | No | Yes | Fair |
| Horgan (2010) (16) | No | Yes | Yes | NA | Yes | Yes | Yes | Yes | Yes | Good |
| Ryou (2009) (30) | Yes | No | Yes | NA | Yes | No | No | No | Yes | Poor |
| Gallo (2016) (17) | Yes | Yes | Yes | NA | Yes | No | Yes | Yes | Yes | Fair |
| Buttelmann (2015) (31) | Yes | Yes | Yes | Yes | Yes | Yes | Yes | Yes | Yes | Good |
| Thompson (2012) (36) | Yes | Yes | Yes | NA | Yes | Yes | No | Yes | Yes | Fair |
| Heylen (2011) (27) | No | Yes | Yes | NA | Yes | No | Yes | No | Yes | Fair |
| Patel (2017) (32) | Yes | Yes | Yes | NA | Yes | No | Yes | Yes | Yes | Fair |
| Tsai (2019) (22) | Yes | Yes | Yes | NA | Yes | Yes | Yes | Yes | Yes | Good |
| Catalano (2007) (33) | Yes | Yes | Yes | NA | Yes | Yes | Yes | No | Yes | Good |
| Loewen (2008) (25) | No | Yes | Yes | NA | Yes | No | Yes | No | Yes | Fair |
| Jirapinyo (2016) (30) | Yes | Yes | Yes | NA | Yes | Yes | Yes | Yes | Yes | Good |
| Thompson (2013) (27) | Yes | Yes | Yes | NA | Yes | Yes | Yes | Yes | Yes | Good |
| de Moura (2019) (39) | No | No | No | NA | Yes | No | Yes | No | No | Poor |
| Kumar and Thompson (2014) (37) | Yes | Yes | Yes | NA | Yes | No | Yes | Yes | Yes | Fair |
| Kumar and Thompson (2016) (36) | Yes | Yes | Yes | NA | Yes | Yes | Yes | No | Yes | Good |
| Jirapinyo (2013) (31) | Yes | Yes | Yes | NA | Yes | No | Yes | No | Yes | Fair |
| Vargas (2018) (29) | Yes | Yes | Yes | NA | Yes | Yes | Yes | Yes | Yes | Good |
| Baretta (2015) (19) | Yes | Yes | Yes | NA | Yes | Yes | Yes | No | Yes | Fair |
| Moon (2018) (18) | Yes | Yes | Yes | NA | Yes | No | Yes | Yes | Yes | Fair |
| Riva (2017) (26) | Yes | Yes | Yes | NA | Yes | Yes | Yes | Yes | Yes | Good |
| Eid (2017) (34) | No | No | Yes | NA | Yes | No | Yes | No | Yes | Poor |
